# Increasing HIV testing among hard-to-reach groups: examination of RAPID, a community-based testing service in Queensland, Australia

**DOI:** 10.1186/s12913-017-2249-5

**Published:** 2017-04-28

**Authors:** Allyson J. Mutch, Chi-Wai Lui, Judith Dean, Limin Mao, Jime Lemoire, Joseph Debattista, Chris Howard, Andrea Whittaker, Lisa Fitzgerald

**Affiliations:** 1 The School of Public Health, The University of Queensland, Brisbane, Queensland Australia; 2 Centre for Social Research in Health (CSRH), University of New South Wales, NSW, Australia; 3 Queensland Positive People, Brisbane, Queensland Australia; 4 Metro North Public Health Unit, Queensland Health, Brisbane, Queensland Australia; 5 School of Social Sciences, Monash University, Melbourne, Victoria Australia

**Keywords:** HIV testing models, STI, Treatment as prevention, Peer led, Rapid testing

## Abstract

**Background:**

The success of ‘treatment as prevention’ (TasP) to control HIV relies on the uptake of testing across priority population groups. Innovative strategies including; rapid HIV testing (RHT) in community and outreach settings, engaging peer service providers, and not requiring disclosure of sexual history have been designed to increase access. This paper reports on the implementation of ‘RAPID’, a community-based testing program in Queensland, Australia that employs these strategies to increase access to testing.

**Methods:**

Service data, including client registration forms and a satisfaction survey from all clients attending RAPID between August 2014 and July 2015 were analysed.

**Results:**

In 2014/2015 1,199 people attended RAPID to receive a free HIV test. The majority were urban-based gay men. 17.1% were first-time testers and 20.1% of participants were not eligible to access Medicare, Australia’s universal health care scheme.

**Conclusions:**

RAPID’s evidence-based strategies appear to facilitate access to HIV testing, particularly among those who have never tested before; however the implications for the ongoing treatment and care of people ineligible for Medicare, who test positive to HIV warrants careful consideration.

**Electronic supplementary material:**

The online version of this article (doi:10.1186/s12913-017-2249-5) contains supplementary material, which is available to authorized users.

## Background

Early diagnosis and rapid initiation of antiretroviral therapy (ART) is a key strategy in the control of the global HIV/AIDS epidemics [1]. Earlier access to testing for HIV and subsequent uptake of ART can improve health outcomes of people living with HIV (PLHIV), potentially eliminating the risk of HIV transmission and reducing HIV notification rates at a population level [2]. The Joint United Nations Programme on HIV/AIDS (UNAIDS) now considers regular testing and early treatment uptake critical components of the ‘treatment as prevention’ (TasP) strategy for HIV risk reduction [3].

The success of TasP relies on the uptake of testing and treatment across priority population groups in the community [4, 5]. Over the last decade, some countries have seen an increase in HIV testing rates [6], but within Australia rates have remained relatively constant with less than two-thirds of gay men meeting current minimum recommendations to test every 12 months [7]. Beyond gay communities, rates of HIV testing among groups that have traditionally been hard-to-reach with conventional venepuncture testing [8], including young people and people from culturally and linguistically diverse backgrounds, are largely unknown [9].

Many barriers undermine the uptake of HIV testing at both the individual (e.g., fear, stigma, perceptions of risk, embarrassment in talking with health professionals) and health service level (e.g., testing location, wait time for results, cost) [8, 10–13]. In response, researchers and service providers have introduced and evaluated innovative approaches and strategies designed to facilitate access to testing. These include the adoption of rapid HIV testing (RHT) technology, use of community and outreach settings for testing, use of peer services, and establishing testing services that do not require the disclosure of sexual history [8, 10, 14]. The following paper reports on the implementation of ‘RAPID’, a novel RHT program developed in Queensland, Australia that has employed a mix of the above-mentioned strategies to increase access to HIV testing.

### Design and characteristics of the RAPID program

In Queensland, and across Australia, the incidence of HIV has continued to increase with the rate of HIV diagnosis per 100,000 rising from 4.4 to 5.1 between 2009 and 2013: in 2013 there were 236 new cases of infection [15]. In a commitment to TasP, the Queensland State Government adopted RHT technology in 2013, following its approval for use in Australia in 2011 [16, 17]. Community-based testing services such as ‘Testing Point’ established by the Queensland AIDS Council, and the ‘RAPID’ testing program, established in August 2014 under the auspice of Queensland Positive People (QPP) in partnership with the HIV Foundation of Queensland, have been instrumental in the roll out of RHT in Queensland. Over half the RHT performed in Queensland in the first quarter of 2015 were conducted at community sites through programs such as RAPID [18].

In comparison with conventional venepuncture testing which requires a trained clinician to take a blood sample and forward it on for laboratory testing, RHT is a cost effective method of testing [19] that produces results in around 20 min at the point of care without the need for clinical supervision or laboratory analysis [20]. Studies comparing RHT with standard testing have found most participants prefer RHT, identifying it as more comfortable, convenient and less stressful [14, 21–23]. Table one provides a summary of the comparisons between community-based RHT services, such as RAPID and clinical services offering conventional HIV testing. Table 1

**Table 1:** Comparisons between community-based RHT and clinical services offering conventional HIV testing in Queensland

	Community-based Peer Testing Services e.g., RAPID	Public Sexual Health Service (PSHS) or Primary Care Provider
HIV test available	RHT	Conventional whole blood sample testing (RHT available in some PSHS)
Who performs HIV pre-test information and test	Trained Peer Testing Facilitator (LGBTQI+ people)	Clinician (e.g., Specialist Sexual Health/HIV Registered Nurses (RN), Sexual Health Physician/HIV Specialist or GP)
Results Waiting time		
RHT	In-house	In-house
Conventional		Off-site at public pathology service
Confirmatory HIV testing	If the test is RHT reactive –conventional HIV test performed by Trained Peer Testing Facilitator and specimen sent to private pathology service for analysis	Analysis performed on initial whole blood sample
Confirmed HIV positive	Referred off-site to PSHS or GP of clients choice for HIV care^b^	HIV care provided on-site^b^
Appointment/Walk-in	Walk-in	Appointment +/− walk-in for symptomatic
Location	Community sites, SOPVs, LGBTQI+ events	Clinics and GP practices
Cost of service to clients	Free	PSHS free, GP may require a co-payment in addition to Medicare^a^ fee
Cost to Health System	Cost effective population testing, but more expensive for HIV reactive results because requires additional test	Less cost effective for population screening than RHT, but for those receiving a positive result it is cheaper than community-based RHT
Pre-test information and sexual health history	Often do not take full history, but provide education while waiting for results	Full sexual history generally standard practice

+/− indicates RHT is only offered at some GP/PHC services offer RHT

^a^Medicare is the Australian Governments universal public health insurance scheme

^b^In Australia Sexual Health Physicians, HIV Specialists and GPs providing HIV care are required to be authorised to prescribe antiretroviral medication (Commonly referred to as s100 Prescribers)

RAPID’s service model draws on an emerging evidence base that indicates locating RHT in non-clinical, community and outreach settings (e.g., shopfronts during festivals, sex on premise venues (SOPV)) can increase access among people who have never tested or tested infrequently [13, 21, 22, 24–26]. Concerns have been raised over issues of confidentiality and professionalism when testing in SOPVs [11, 13], but evidence of high levels of user satisfaction [11] and increased access for hard-to-reach groups [13, 26–29], particularly in comparison with conventional testing located in clinical settings, were central drivers for RAPID.

Lornec and colleagues’ systematic review of testing preferences highlighted the need for testing services that are: “community-based, friendly, culturally competent, gay positive and that normalised sexuality and STD/HIV testing” [10]. In line with this, RAPID developed a peer-based service that relies on trained community members to provide testing and education services. Evidence suggests this can be an effective strategy designed to create a friendly and comfortable environment [30, 31] that may increase engagement among those who have not previously tested or those who have felt stigmatised in clinical settings [25, 26, 32]. The peer model also allowed RAPID to step beyond traditional clinical assessment methods, which emphasise sexual behaviour and history taking, to further reduce barriers and ‘normalise’ HIV testing [33, 34].

In sum, RAPID adopted all of the strategies identified by current best evidence to provide an accessible service designed to increase HIV testing, particularly among those who had never tested for HIV. The following paper examines the characteristics of people who attended RAPID and tested for the first time to consider the extent to which RAPID met its objectives.

## Methods

This paper analysed service data gathered from all clients attending RAPID between August 2014 and July 2015. The data gathered by RAPID included a registration form, client satisfaction survey and an attendance log. The registration and client satisfaction forms were developed based on RAPID’s philosophy of ‘testing without the full Q&A’, thus questions were kept to a minimum (20 items). The self-complete registration form included age, gender, sexual orientation, country of birth, Medicare card (yes/no), postcode, and how the client heard about RAPID Additional file 1.

The client satisfaction survey, self-completed at the end of the visit, collected HIV testing history and reason for testing on this occasion, and satisfaction with the RAPID service. The attendance log recorded information on testing venue (RAPID main clinic or SOPV), people who returned for one or more tests, and people who were HIV reactive.

Ethics approval for the study was obtained from the Metro North Hospital and Health Service and The University of Queensland Human Research Ethics Committee (Approval No. 2015001063).

### Data analysis

Data were analysed using SPSS^TM^ software (Version 22, Statistical Software for Social Sciences, IBM, Chicago). Descriptive statistics (means, standard deviations, frequencies, per cents) were performed on all client characteristic variables. The sample was then divided and data summarised and compared based on whether a person had ever tested for HIV. A significance level of *P* < 0.05 was used for all tests.

## Results

One thousand four hundred eighty-four RHTs, involving 1,199 people, were conducted by RAPID between August 2014 and June 2015. The following results are based on the person’s first visit to RAPID or its outreach services at SOPVs and excluded those attending for an additional test(s) (Table 2). The majority of clients were men (95.4%) who identified as gay or bisexual (85.1%). The mean age was 34.9 and most (81.1%) lived in the greater Brisbane area. Over half (59.8%) were born in Australia with the remainder representing over 70 different countries. One in five people (20.1%) attending RAPID were not eligible to access Medicare, Australia’s universal health care scheme that covers core health service costs. The majority of these individuals were born overseas (91.7%:221).

**Table 2:** Characteristics of all RAPID Clients and First-time Testers

	All RAPID Clients *n* = 1199 (%)^a^	First-time Testers *n* = 191 (17.1%)
Gender		
Male	1144 (95.4)	170 (89.0)
Female	53 (4.4)	21 (11.0)
Transgender	2 (0.2)	0
Age		
Mean (Standard Dev)	34.9 (12.9)	30.8 (13.6)
Range	17–83	18–83
Sexuality		
Gay/Bi-sexual	1017 (85.0)	133 (69.6)
Straight	179 (15)	58 (30.4)
Indigenous	26 (2.2)	2 (1.0)
Country of Birth		
Australia	717 (59.8)	96 (50.3)
Other	481 (40.2)	95 (49.6)
Residential location		
Urban	935 (81.1)	147 (79.5)
Regional/Rural	129 (11.2)	25 (13.5)
Other	89 (7.7)	13 (7.0)
Testing Venue		
RAPID Clinic	885 (78.2)	149 (80.1)
SOPV	247 (21.8)	37 (19.9)
Ever Tested		
Yes	927 (82.9)	–
No	191 (17.1)	–
Frequency of testing		
Within the last 12 months	619 (55.4)	–
More than 12 months ago	308 (27.5)	–
First time	191 (17.1)	191 (100.0)
Medicare Ineligible	241 (20.1)	57 (29.8)

^a^Not all categories add up to 1199 due to missing data

Twenty-one men (1.8%) received a reactive HIV test result which was confirmed with subsequent clinical testing. The majority attended the main RAPID testing site with four (1.6%) people tested at an SOPV. Most of the people who received a reactive result identified as gay or bisexual (95.2%:20), living in the greater Brisbane area (76.2%:16) and approximately half were born in Australia (47.6%:10). Frequency of testing was poorly reported amongst the sub-sample of people testing positive for HIV with only four people indicating that they had been tested in the last year. Six (28.6%) people who tested positive were Medicare ineligible.

### First-time testers

One hundred ninety-one (17.1%) people had never previously tested for HIV and were tested for the first-time at RAPID. The majority of first-time testers were men (89.0%: 170). First-time testers were significantly younger (X = 30.86 years) than previous testers (X = 35.94 years) (t = −4.76 (261) *P* < 0.05) and 40% of women visiting RAPID were testing for the first time compared with only 14.9% of men (*X* ^2^ = 22.85 (1) *P* < 0.05). The majority of first-time testers visited the main testing centre; there was no association between testing venue and having ever tested for HIV. First-time testers were significantly more likely to be Medicare ineligible (29.8%: 57) compared to those who had previously tested (17.2%:159) (*X* ^2^ = 16.36 (1), *P* < 0.05). People who had never tested were significantly more likely to identify as straight (30.4%: 58) when compared to people who had tested previously (11.4%:106) (*X* ^2^ = 45.24 (1) *P* < 0.05). There was no association between residential location and ever tested.

### Gaining information about RAPID

Nearly a third (29.6%: 338) of RAPID clients gained information about the service through Google and nearly half (44.1%:82) the people who received their first HIV test obtained information about the service through this channel (Table 3). Word of mouth and access through the outreach service at SOPVs were also common sources of information about RAPID.

**Table 3:** Source of information about RAPID and reasons for testing

	All RAPID Clients (*N* = 1199)	First-time testers *n* = 191 (17.1%)
Hear about the service		
Google	338 (29.6)	82 (44.1)
SOPV	270 (23.7)	37 (19.9)
Word of mouth	209 (18.3)	33 (17.7)
Dating site/App	190 (16.7)	18 (9.7)
Press	81 (7.1)	5 (2.7)
Facebook	53 (4.6)	11 (5.9)
Reason for testing^a^		
Condomless sex	371 (30.9)	72 (37.7)
Because the results are available in 20 mins	367 (30.6)	55 (28.8)
To have my regular 3/6/12 month test	201 (16.8)	^b^
Recommended by a friend or other person	141 (11.8)	38 (19.9)
About to enter/finish a relationship	120 (10.0)	23 (12.0)
Dating App Advertisement	119 (9.9)	21 (11.0)
Media Coverage about RAPID or HIV	75 (6.3)	15 (7.9)
Contact with an HIV outreach worker	73 (6.1)	10 (5.2)
Someone I know has been diagnosed	75 (6.3)	15 (7.9)
Perceptions of RAPID^c^ Comfort with	(strongly agree/agree)	(strongly agree/agree)
More likely to test more frequently at a peer based service	990 (88.6)	165 (86.4)
Availability of RHT will increase frequency of testing	1118 (87.3)	159 (83.0)
Overall Satisfaction with a peer based service	1105 (98.8)	191 (99.0)

^a^Respondents could select more than one option

^b^Not a valid response for first-time testers

^c^Not all categories add up to 1199 due to missing data

### Reasons for testing

Condomless sex was the most common reason people identified for accessing the service: nearly one-third of all RAPID clients (30.9%: 338) and over a third (37.7%:72) of first-time testers accessed RAPID for this reason. The speed and convenience of the RHT were also identified as key reasons for attending RAPID. Recommendations from friends (11.8%: all clients; 19.9%: first-time testers) and entering or exiting a relationship (10%: all clients; 12%: first-time testers) were identified by some participants as key reasons for having a test.

### Perceptions of RAPID

Participants were asked to complete a number of questions related to their satisfaction with the RAPID service, particularly in comparison with conventional venepuncture testing. Of those who had previously tested, the vast majority (89.8%: 832) felt much more comfortable or more comfortable receiving an RHT at RAPID compared to conventional testing. The overwhelming majority (82.5%: 765) of previous testers also described the experience of receiving an RHT at RAPID as much less stressful and anxiety provoking when compared to conventional testing. The majority of participants, including previous testers and those who had never tested, said they were more likely to increase the frequency of testing given the availability of RHT testing, and access to a peer-based service such as RAPID. The overwhelming majority of participants were satisfied with the RAPID service (Table 3).

## Discussion

This paper highlights the diverse characteristics of people who attended the RAPID testing service during its first year of operation and the overwhelming support and satisfaction of clients accessing the service. The adoption of evidence-based strategies designed to enhance access, including the use of RHT in a convenient, peer-led, community-based service, appear to have supported the Service to achieve its aims of investing in the early detection of HIV and enhancing access to HIV testing, particularly among those who have never tested before.

In highlighting the benefits of the RAPID program, it is clear that drawing conclusions about the Service’s effectiveness is limited by the study’s reliance on service data and the absence of a control group; however comparisons with other testing services may serve to illustrate the benefits of RAPID’s approach. Specifically, the rate of first-time testing at RAPID was consistent with other similar innovative community-based testing services recently established in Sydney and Melbourne [26, 35], but exceeded rates identified in general sexual health clinic samples using the same RHT technology (e.g., 10.1% reported by Conway and colleagues [36]). This comparison between community-based services such as RAPID, and clinic-based services suggests community offers an effective approach for increasing access to testing, particularly among those who have never tested.

Beyond the strategies employed by RAPID to increase access, a number of factors appear instrumental in increasing awareness and uptake of the Service. The first relates to the way in which people identified RAPID. Nearly a third of all clients, and almost half of first-time users, used the internet to identify a site for testing. A substantially smaller number of people, particularly among first-time testers, accessed RAPID after seeing an advertisement on a dating app. These results highlight the importance of a strong on-line presence, but consistent with broader evidence, suggest limited value in relying on advertising through dating apps as a way to increase testing uptake [37, 38].

Consistent with a broad body of evidence [39–42], the reasons for testing appear to be strongly linked to perceptions of risk of HIV arising from a specific event or incident. Nearly a third of RAPID clients and over a third of those who were testing for the first time, visited RAPID because they had recently had condomless sex, while fewer than 20% were attending to have their regular test. Immediate perceptions of risk continue to drive testing behaviours within the community [10, 36, 42], while efforts to promote regular testing, in line with the aims of TasP, appear to still have some way to go. Notably, the ability to receive test results quickly and undergo a test that was perceived as ‘less stressful’ were prominent reasons for accessing RAPID. This finding clearly supports the Service’s use of RHT technology as a method to increase access and reinforces the investment made by policy makers in Queensland.

A final observation emerging from this examination of RAPID relates to Medicare eligibility. Nearly a third of first-time testers and a third of those who received a reactive HIV test were ineligible for Medicare. Access to Medicare is limited to Australian citizens and permanent residents: temporary residents on a range of visas including; student, working, bridging or spousal visas are excluded [43]. While it is difficult to provide further details of this group due to the limitations of the available RAPID service data, broader evidence suggests this ineligibility often parallels other characteristics that magnify a person’s vulnerability, including constrained financial resources, and social isolation [8, 44]. In providing testing services to all community members, regardless of their Medicare status, RAPID has clearly met its goal of increasing access to testing; however the more serious implications of Medicare eligibility for those who tested positive for HIV, but are unable to access subsidised treatment, are undeniable and their circumstances warrant further detailed investigation. Consistent with TasP, RAPID has endeavoured to increase testing rates, but unless everyone who tests positive, including temporary residents, can also access treatment, the TasP strategy will fall short of its goals [43].

## Conclusions

The current paper provides a valuable insight into the diversity of people accessing the RAPID service. While a more detailed consideration is limited by the data, which were gathered to support the RAPID Service and its delivery rather than to inform a wider research agenda, the findings do provide useful insight into the valuable role a community-based testing service, such as RAPID, can play in enhancing access to HIV testing. However, unless policy makers continue to ensure that an accessible service is also matched by access to treatment the commitment to TasP and achieving its goals may fall short.

## Additional file

Additional file 1: Rapid Client Satisfaction form. (PDF 107 kb)

## Abbreviations

ART: Antiretroviral therapy
PLHIV: People living with HIV
QPP: Queensland Positive People
RHT: Rapid HIV testing
SOPV: Sex on premise venues
TasP: Treatment as prevention’
UNAIDS: United Nations Programme on HIV/AIDS
