# Supplementary material for: Increasing HIV testing among hard-to-reach groups: examination of RAPID, a community-based testing service in Queensland, Australia
Source: BMC Health Serv Res. 2017 Apr 28;17:310. doi: 10.1186/s12913-017-2249-5 (PMC5410036; doi:10.1186/s12913-017-2249-5)
Supplement: Additional file 1: — Rapid Client Satisfaction form. (PDF 107 kb) [file 12913_2017_2249_MOESM1_ESM.pdf]

RAPID

CLIENT SATISFACTION FORM

Please answer the questions below and return to your Peer Education and Testing Officer

1. When were you last tested for HIV?

- ☐ Never
- ☐ Less than a week
- ☐ 1-4 weeks
- ☐ 1-6 months
- ☐ 7-12 months
- ☐ 1-2 years
- ☐ 2-4 years
- ☐ More than 4 years

2. Which of the following best describe your usual testing frequency of HIV testing?

- ☐ Every 3 months (or more often)
- ☐ Twice a year
- ☐ Once a year
- ☐ Less than once a year
- ☐ I have never been tested before

3. Would the availability of rapid HIV testing affect the frequency that you test for HIV?

- ☐ Yes, I would test more often
- ☐ No, I would not test more often
- ☐ Unsure/don't know

4. How likely would you be to test more frequently at a peer-based service, like Rapid?

- ☐ Much more likely
- ☐ More likely
- ☐ Same as a clinical setting
- ☐ Less likely
- ☐ Much less likely

5. How much stress and anxiety did you experience today having a rapid HIV test compared to conventional HIV testing?

- ☐ Much less stress and anxiety
- ☐ Less stress and anxiety
- ☐ Similar stress and anxiety
- ☐ More stress and anxiety
- ☐ Much more stress and anxiety
- ☐ I haven't been tested for HIV before

6. How did the comfort of a finger prick puncture for the rapid HIV test compare with the comfort of having blood drawn from your arm for conventional HIV testing?

- ☐ Much more comfortable
- ☐ More comfortable
- ☐ Similarly comfortable
- ☐ Less comfortable
- ☐ Much less comfortable
- ☐ I haven't been tested for HIV before

7. Overall, I was satisfied with my experience of peer-based rapid HIV testing.

- ☐ Strongly agree
- ☐ Agree
- ☐ Neutral
- ☐ Disagree
- ☐ Strongly Disagree

8. Lastly, why did you decide to come for a test today? *(please tick all that apply)*

- ☐ Had condomless sex
- ☐ Dating app advertisement
- ☐ Have not had a test in more than a year
- ☐ About to enter/finish a relationship
- ☐ Media coverage about HIV or Rapid
- ☐ Contact with HIV outreach worker
- ☐ Shared injecting equipment
- ☐ Regular 3/6/12 month test
- ☐ Because results are in 20 minutes
- ☐ Someone I know has been diagnosed with HIV
- ☐ Recommended by a friend or other person
- ☐ Other ______________________

Thankyou for your time ☺

RAPID

REGISTRATION FORM

Given Name: ______________________ **Surname:** ______________________

Date of Birth: ______________________ **Country of Birth:** ______________________

Postcode: ______________________ **Mobile number:** ______________________

Email address *(optional)*: ____

Do you have a Medicare Number? ☐ Yes ☐ No

Do you identify as: ☐ Male ☐ Female ☐ Trans*

Do you identify as: ☐ Aboriginal ☐ Torres Strait Islander ☐ Both ☐ Neither

Would you like a reminder for your next test? ☐ SMS ☐ Phone ☐ Email

How did you first hear about Rapid?

☐ **Dating site/App** ☐ **Facebook** ☐ **Press**

☐ **Venue** ☐ **Google** ☐ **YouTube**

☐ **Recommendation/Word of mouth**

Please turn over and read the Privacy Statement and Follow Up Protocol. Once read, please show you consent to the information by signing at the bottom of the page.
